# Staying cool: preadaptation to temperate climates required for colonising tropical alpine-like environments

**DOI:** 10.3897/phytokeys.96.13353

**Published:** 2018-04-17

**Authors:** Berit Gehrke

**Affiliations:** 1 Institut für Organismische und Molekulare Evolutionsbiologie, Johannes Gutenberg-Universität, D-55099 Mainz, Germany

**Keywords:** Alpine speciation, biome change, island biogeography, niche conservatism

## Abstract

Plant species tend to retain their ancestral ecology, responding to temporal, geographic and climatic changes by tracking suitable habitats rather than adapting to novel conditions. Nevertheless, transitions into different environments or biomes still seem to be common. Especially intriguing are the tropical alpine-like areas found on only the highest mountainous regions surrounded by tropical environments. Tropical mountains are hotspots of biodiversity, often with striking degrees of endemism at higher elevations. On these mountains, steep environmental gradients and high habitat heterogeneity within small spaces coincide with astounding species diversity of great conservation value. The analysis presented here shows that the importance of *in situ* speciation in tropical alpine-like areas has been underestimated. Additionally and contrary to widely held opinion, the impact of dispersal from other regions with alpine-like environments is relatively minor compared to that of immigration from other biomes with a temperate (but not alpine-like) climate. This suggests that establishment in tropical alpine-like regions is favoured by preadaptation to a temperate, especially aseasonal, freezing regime such as the cool temperate climate regions in the Tropics. Furthermore, emigration out of an alpine-like environment is generally rare, suggesting that alpine-like environments – at least tropical ones – are species sinks.

## Introduction

Latitudinal and elevational gradients (Pianka 1966; Dowle et al. 2013) of plant distribution and richness are wellstudied patterns and appear to result from the tendency of plants to retain their ancestral ecology (Donoghue 2008 and references therein). These patterns are the basis for the establishment of floristic regions, biomes, ecoregions and floristic elements (Cain 1944; Good 1974; Whittaker 1975; Takhtajan 1986; Olson et al. 2001; Sklenář 2011). However, isolation can occur at any scale where there are discontinuities in realised environmental niches, such as between lakes or ponds, inselbergs or mountain tops. Movement between such habitats can be rare, irrespective of their size, location and geographical proximity (see for example Crisp et al. 2009).

**Figure 1.**
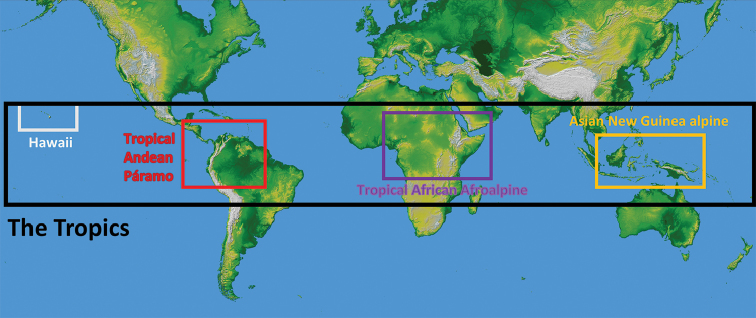
Location of the tropical alpine-like climate regions in the Tropics on a Mercator projection of the world with shaded relief and coloured height based on SRTM data with 1 arc second resolution. Credit: NASA/JPL/NIMA downloaded from http://photojournal.jpl.nasa.gov/catalog/PIA03395. Detailed maps for each region are included in the Suppl. material 3 (figures S3–S6).

Alpine-like or supra-alpine environments, especially those on isolated mountains, are a notable example of such isolation (Hedberg 1965). The alpine-like flora is typically strikingly different from that of the surrounding lowlands. Trees are absent and, in their place, are species with life histories and growth forms that are adapted to grow under harsh conditions that include frequent and lasting freezing temperatures and short growing seasons (Körner 2003). At low latitudes, the contrast between alpine-like conditions found only on the highest mountains (Fig. 1) and the tropical environments that surround them is particularly stark. Plants adapted to high elevations generally cannot survive at low elevations and vice versa. Alpine-like climate regions in general and those of the Tropics in particular, are therefore considered akin to isolated oceanic islands (Smith and Cleef 1988).

It is well established that recruitment via long-distance dispersal is of paramount importance to the evolution of both oceanic- and mountaintop ‘sky’- islands (Alsos et al. 2007; Weigelt and Kreft 2013) and that recruitment was followed only in some cases by *in situ* radiations (MacArthur and Wilson 1967; Carlquist 1974; Baldwin and Sanderson 1998; Ackerly 2003; Cameron et al. 2013). However, the relative importance of these processes remains poorly understood but is significant, especially for the prediction of changes in species diversity in the light of climate change.

Here, the origin of species is investigated in tropical-alpine floras across the world, comparing South American tropical (Supra-)Páramo, the tropical Afroalpine in Africa, tropical alpine-like Mount Kinabalu in Southeast Asia and high elevation areas on the Pacific Ocean Islands of Hawaii (Fig. 1). The following hypotheses are tested: i) the importance of *in situ* speciation in tropical alpine-like areas has been underestimated, ii) the impact of dispersal from regions with alpine-like environments is large compared to that of immigration from other regions and iii) emigration out of an alpine-like environment is generally rare.

## Methods

### Area

Tropical alpine-like areas are defined as regions located above the natural high-altitude treeline, that is the upper limit of tall upright, woody life forms, within 23°26'N, 23°26'S (Smith and Young 1987; Smith and Cleef 1988; Smith 1994; Rundel et al. 1994; Körner 2003, Nagy and Grabherr 2009). Mountains, especially those in the Tropics, show distinct changes in life conditions with elevation and the rise of isotherms as one moves towards the equator. The location of the natural treeline is considered here to be at about 4000 m, as long as annual precipitation is >250 mm (Körner and Paulsen 2004; Körner 2012). The upper altitudinal limit commonly extends to between 4600 and 5000 m (Smith and Young 1987; Luteyn 1999).

True tropical alpine-like environments are characterised by a mean daytime soil temperature of about 6 °C (Körner 2011) but also by recurring events of frost especially at night with a small amplitude of climatic differences throughout the year. For East Africa, this has been described as “summer every day and winter every night” (Hedberg 1965) which also leads to the absence of a distinct growing or flowering ‘season’ as most plants can photosynthesis and have been reported as flowering throughout the year.

Other authors have used a different definition of the term “tropical alpine” or tropicalpine by only considering the present day upper tree limit as the cut-off line, therefore including areas above ~3500 m (Sklenář et al. 2011; Gehrke and Linder 2014) or even as low as 2000 m in various tropical islands (Leuschner 1996). Some authors tried to avoid the term alpine and referred to these regions using terms such as Páramo and Afro-alpine (see Smith and Young 1987, for a detailed review on terms) or even just summit zone or high elevation areas (Smith 1980, 1994).

Due to the high frequency of plants growing in micro niches along a wide elevational amplitude and the mosaic-like patterns of elevational plant zones, a strict definition of the term ‘alpine-like’ is used and only includes plants in areas above 3800 m with a clear diurnal rather than seasonal pattern of vegetation growth and flowering (Table 1; Fig. 1). Therefore, the tropical alpine-like areas are defined here as follows: they are regions located above the natural high-altitude treeline, within 23°26'N, 23°26'S, characterised by a mean daytime soil temperature of about 6 °C (Körner 2011) and by recurring events of frost especially at night with a small amplitude of climatic differences throughout the year. To avoid including too many species that barely reach into the tropical alpine-like regions, a lower limit of 3800 m has been chosen.

**Table 1.** Overview of high tropical alpine-like regions as used in this study.

Region	Andean Páramo	Afroalpine	Malesia and New Guinea	Hawaii
Estimated area (km^2^)	23,452^1^	4510	2000	<500?
Number of species	3026	521	1.118	13
Number of genera	449	191	226	10
Typical alpine species	998	163	~400/67^2^	13
Alpine species used	668	145	18	12
% endemism	60^3^	67^4^	60^5^	100
Species turnover^6^	highest	lowest	medium	n.a.
Largest genus (no. species in alpine/ region/ genus)	*Draba* (27/70/370)	*Senecio* s.str. (32/>130/~1000)	*Rhododendron* (21/219/>1000)^8^	Silversword alliance (3/28/28)

^1^estimated based on “altoandino” (Josse et al. 2009), ^2^number of species recorded to occur in the alpine zone on Mt.Kinabalu, ^3^Nagy and Grabherr 2009, ^4^Gehrke and Linder 2014, ^5^estimated here, ^6^beta diversity (Sklenář et al. 2014) based on the analysis of seven mountains in each region, ^7^*Draba* is listed here as largest genus despite 37 *Senecio* species recorded in the high Andean Páramo and 68 in the wider Páramo (Luteyn 1999) because the number of species belonging to a monophyletic *Senecio* s.str. is not clear, ^8^insufficient data.

**Northern (Supra-)Páramo**

Following the above definition, the majority of areas with a tropical alpine-like environment are located in the Americas, i.e. in the Andes between Venezuela and Peru and residual Páramo ecosystems in Costa Rica (highest point: 3810 m), with the exclusion of the drier regions at higher altitudes in Mexico (Nagy and Grabherr 2009) and the drier Puna and Jalca regions in the Central and southern Andes (Luteyn 1999). Some authors use the term Supra-Páramo for this (Lauer 1981). These regions are by far the largest in area (probably more than 90% of the total tropical alpine area, Jacobsen 2008; Sklenář et al. 2011, 2014) as well as the most species rich even when correcting for area size (Gehrke and Linder 2014).

**Africa**

In Africa, most alpine-like environments are located in the eastern mountain ranges of the continent (White 1983; Gehrke and Linder 2014) as well as a smaller area on volcanic Mount Cameroon (4095 m, Letouzey 1985). In the tropical Afroalpine, mountain ranges are well separated from each other, mostly by large tracks of lowlands (Hedberg 1951; Chala et al. 2017).

**Malesia and New Guinea**

New Guinea harbours the most extensive alpine-like environment in South-east Oceanic Asia in addition to scattered areas on a number of Indonesian islands and in Malaysia including Mt. Kinabalu on the island of Borneo (Smith 1994; Sklenář et al. 2014). Unfortunately, floristic and phylogenetic data for plants in the New Guinea alpine region are even scarcer than that for plants from the northern Andean Páramo or tropical Afroalpine. Only data on the relatively well-studied plants from Mt. Kinabalu in the northern part of the island of Borneo are therefore included.

**Hawaii**

Hawaii is the most isolated and most northerly area investigated. The absence of a strong seasonal climate is likely due to the moderating effect of the surrounding ocean. Hawaii is situated close to the northern limits of the Tropics (max. elevation 4100 m) and only harbours a small high alpine-like environment (Leuschner 1996). In general, most recent phylogenetic evidence suggest that tropical-alpine like climate developed only fairly recently around the world in the Plio-Pleistocene (Madriñán et al. 2013; Gehrke et al. 2014).

### Character coding

All analyses were undertaken at the species level, ignoring sub-specific taxa even if these were confined to tropical alpine-like areas. The origins of species in the topical alpine-like environments were coded as *in situ* speciation versus immigration. The former includes all species in the tropical alpine-like that have originated from an ancestor in the tropical alpine of the same area, the latter includes those with a most recent common ancestor inferred to have been elsewhere. For species that originated by immigration, the author inferred whether they originated from areas with an alpine-like climate, a tropical climate or otherwise.

Initially, the author attempted to draw finer distinctions within the non-alpine and non-tropical category, such as distinguishing temperate climate regions located in the Tropics or elsewhere. However, this proved to be impossible due to low resolution, low sampling and inadequate support in most phylogenetic reconstructions. The three categories of alpine-like-, tropical or other climate were therefore used. It proved extremely difficult to discern between biome change and *in situ* speciation for species that have a widespread distribution, as phylogeographic data would be necessary to exactly place their origins and such data are not available.

Only a minority of species, found in the tropical alpine-like are exclusively confined to these regions, even if they predominantly occur there (i.e. they are regarded as typical alpine elements). For example, 60 percent of Afroalpine species (Gehrke and Linder 2014) and 85 percent of those on Mt. Kinabalu also occur elsewhere. Within the Andes, this value seems to vary more widely and it is particularly high on the western slopes of the Peruvian Andes, where alpine species merge gradually with species of montane pasture, savannah or desert (Smith 1994). Only species that grow most frequently above 3800 m and higher were therefore included, i.e. those that either have most of their distribution above 3800 m or that are able to grow above 4200 m. When molecular phylogenetic evidence was unavailable, the most recent taxonomic classification was used as a hypothesis of evolutionary relationships, in the knowledge of the limitations of this approach, particularly due to morphological convergence. For detailed coding examples and the full list of taxa and their coding see Suppl. material 1: Methods.

## Results

**Figure 2.**
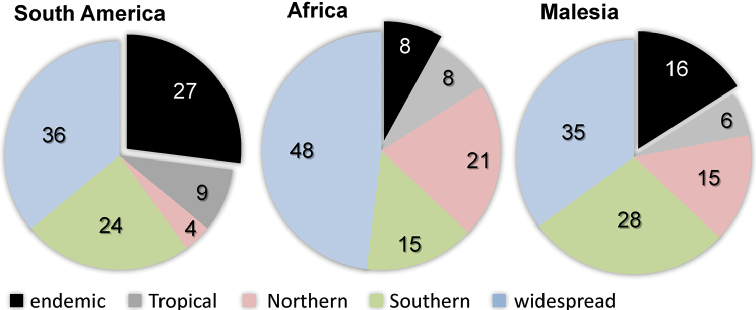
Relative contribution of *in situ* speciation and immigration to species richness in selected tropical alpine regions (pie charts on the left). Blue: *in situ* speciation, green: colonisation, light blue: uncertainty regarding *in situ* speciation, light green: uncertainty about colonisation. In the right pie charts, colonisation is further decoupled into species derived from other regions with alpine-like climate (black) and species that originated by colonisation from a different biome (red). Uncertainty is indicated by grey.

The relative contribution of *in situ* speciation versus immigration differs between the different tropical alpine regions (Fig. 2). *In situ* speciation seems to have been rare on Mt. Kinabalu and Hawaii (one to two or no recorded *in situ* speciation events respectively), while it contributed between 199–392 species to the Andean Páramo (30–59%) and 48–61 to the Afroalpine species (29–37%).

In total, colonisations from other environments accounted for 318–428 (36–75%) of all species in the Páramo, 67–74 (51–61%) in the Afroalpine, 8–9 (83–94%) on Mt. Kinabalu and 10 (91–100 %) on Hawaii. Species originating from other alpine-like biomes, i.e. alpine-like regions outside the Tropics, account for 46–102 species in the Páramo (7–15% of all species or 12–27% of colonisation events), 16–25 in the Afroalpine (10–15% total or 15–23%), 7–8 on Mt. Kinabalu (39–44% total or 42–48%), and 1 on Hawaii (see Suppl. material 2: Results). There was no evidence for direct transitions between tropical climate environments.

Despite the lack of phylogenetic evidence for direct dispersals between alpine-like regions, there are 52 genera found in both the Northern Andean Páramo (Luteyn 1999, Sklenář 2011 including 449 genera) and the tropical Afroalpine (sensu Gehrke and Linder 2014 including 191 genera). Shared genera are:

• **Holarctic element** (north temperate and Mediterranean distribution, 12 of a total 41 of genera in the Páramo s.l.): *Astragalus*, *Bartsia*, *Cerastium*, *Clinopodium*, *Erigeron*, *Lathyrus*, *Lithospermum*, *Potentilla*, *Salvia*, *Saxifraga*, *Silene*, *Vicia*.

• **Wide temperate element** (temperate and cool regions of both hemispheres, 35 of a total of 70 genera in the Páramo s.l.) : *Agrostis*, *Alopecurus*, *Aphanes*, *Bromus*, *Callitriche*, *Cardamine*, *Carex*, *Crassula*, *Cynoglossum*, *Danthonia*, *Deschampsia*, *Epilobium*, *Festuca*, *Galium*, *Geranium*, *Gnaphalium*, *Hypericum*, *Isolepis*, *Juncus*, *Limosella*, *Luzula*, *Myosotis*, *Poa*, *Polypogon*, *Potamogeton*, *Ranunculus*, *Rubus*, *Rumex*, *Senecio*, *Sedum*, *Stellaria*, *Trisetum*, *Valeriana*, *Veronica*, *Viola*.

• **Cosmopolitan** (3 of a total of 17 genera in the Páramo s.l.): *Euphorbia*, *Hydrocotyle*, *Lobelia*.

• **Wide tropical** according to Sklenář et al. 2011: *Conyza*, *Eragrostis*, *Eriocaulon*.

The author did not include either *Lithospermum* or *Euphorbia* in the analyses of origin because neither meet the stricter definition of alpine-like outlined in the methods, i.e. they do not occur in true alpine-like conditiouns above 3800 m.

In general, the vast majority of plant lineages present in tropical alpine-like environments are also present at lower elevations (see Suppl. material 3, Figures).

## Discussion

Several studies have assessed the origin of plant lineages in one or more tropical alpine-like regions, mostly summarising their data by genus (Engler 1879, 1892; Hedberg 1965; Smith 1975; Smith and Cleef 1988; Beaman and Beaman 1990; Nagy and Grabherr 2009; Sklenář et al. 2011, 2014) and more rarely using phylogenetic evidence (Sklenář et al. 2011). Most studies reflect a strong notion of (broad) climatic niche conservatism within genera. However, genera are a poor proxy for independent evolutionary lineages in the tropical alpine, as shown by the various African taxa derived from multiple independent colonisation events from Eurasia (Assefa et al. 2007; Gehrke and Linder 2009), Southern Africa (Galley et al 2007' Kandziora et al. 2016) or lower elevations (Knox 2004; Galbany-Casals et al. 2014). Prominent examples of these repeated colonisations of the Afroalpine are the giant lobelias and giant senecios, which have both been postulated to have originated by repeated upward adaptation to alpine-like environments (Knox 2004). Similarly, in *Halenia*, the majority of species in the high elevation Páramo are the result of repeated immigrations, in this case from Central America (von Hagen and Kadereit 2003). However, the phenomenon of repeated immigration is better understood for regions elsewhere, for example the alpine-like tundra environment on the isolated island of Svalbard in the Northern Sea that has been colonised by many plant groups several times, even within individual species (Alsos et al. 2007).

It has been suggested that much of the plant diversity in tropical alpine regions originated outside the tropics (Hedberg 1961; Smith 1975; Smith and Cleef 1988, Fig. 3). For example, Sklenář et al. (2011) calculated that around half of all lineages in the Andean Páramo are of temperate origin (in a geographical, rather than climatic, sense). Similar estimates have been made for tropical Afroalpine plants (Hedberg 1961). All this would suggest a high proportion of long distance dispersal from similar cool climates, a pattern notably lacking in the results reported here (i.e. the small reported proportion of direct alpine or alpine-like to alpine-like dispersals). In fact, alpine to alpine long-distance dispersal seems rare compared to biome shifts, irrespective of the degree of isolation by distance and despite the numerous genera in common (e.g. Páramo and Afroalpine share about 50 of 449 genera in the Páramo and 191 in the Afroalpine, see Suppl. material 2: Results). An intuitive explanation for limited dispersal between tropical alpine habitats would be the extensive intervening distances and the small overall areas involved. The greater importance of alpine-like regions as a source of species on Mt. Kinabalu probably reflects discontinuity within the New Guinea alpine region and it might therefore be better compared to single mountains within other such areas. Hawaii, as the most extreme case of low importance of alpine-like source regions, is geographically isolated, which probably limits colonisation success. However, the low proportion of alpine-like immigration for the Páramo cannot be explained in the same way. The Andes form a more or less continuous chain of high elevation, cool-temperate environments ranging from northern Colombia to southern Chile. Nevertheless, many Andean plant lineages show – even at lower elevations – limited dispersal between the Northern and Southern Andes with independent diversifications in each region (e.g. *Puya*: Jabaily and Systma 2012, *Ourisia*: Meudt and Simpson 2006, *Azorella*: Nicolas et al. 2012). This may suggest that transitions from a seasonal to aseasonal freezing climate (i.e. from more southern Andean temperate climate regions to more northern Andean tropical alpine-like environments) might require more adaptations at different life stages than has previously been recognised (Neuner 2014).

**Figure 3.**
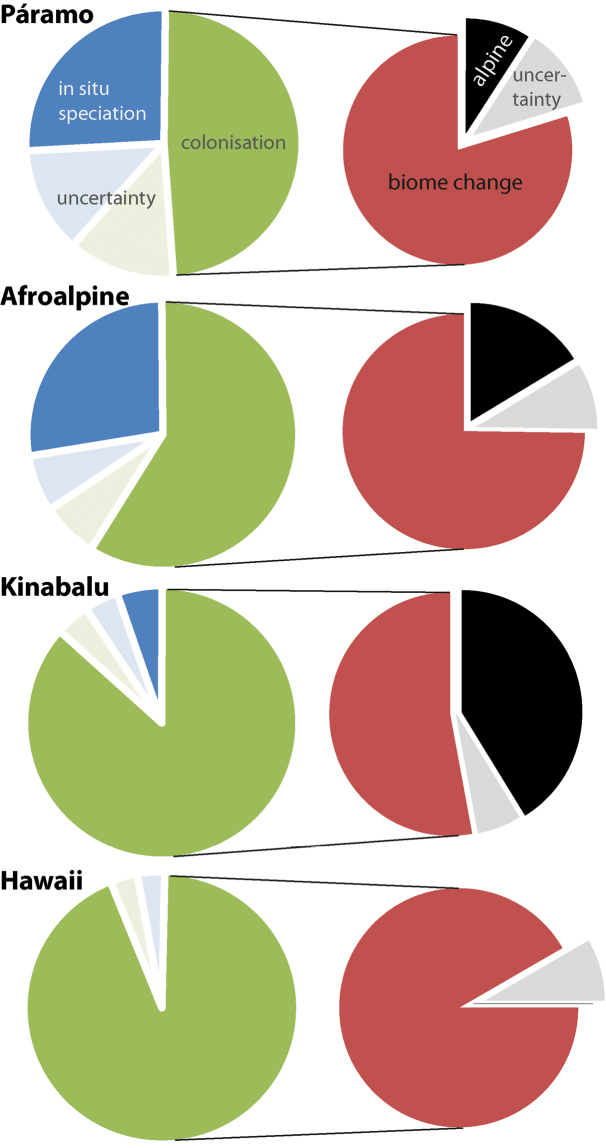
Proportion of plant elements in tropical alpine regions based on generic distribution patterns according to Smith and Cleef (1988).

There is a striking lack of evidence for any direct establishment of warm-tropical species in tropical alpine-like climates. This reflects a similar lack of evidence in numerous plant lineages and even families in general for transitions from tropical to temperate climates. As such, freezing temperatures seem to be a major factor limiting species distributions in (sub-) tropical high mountain ecosystems (Neuner 2014). Overall, it seems that most plants that colonise the true tropical alpine-like aseasonal ‘summer every day and winter every night’ climatic regime first establish at lower elevations. Additionally, there is no unequivocal evidence for long-distance dispersal of tropical alpine plants to cool temperate zones elsewhere. This is similar to the pattern revealed in Southern Hemisphere biome transitions (Crisp et al. 2009), in which the alpine biome was the most common destination of biome transitions but rarely the source of them. This might, at least in part, be explained by the young age of alpine-like regions and the relatively small area they occupy.

An important factor in reaching these conclusions is a consistent differentiation between alpine-like climate regions and those at lower elevations, a distinction that is often blurred in literature. For example, a commonly cited Páramo group is the Asteraceae subtribe Espeletiinae, consisting of over 140 species distributed across Venezuela, Colombia and Ecuador in both Páramo and in montane forest (Rauscher 2002, Sklenář et al. 2011). Interestingly, of the 140 species, only 61 are reported to occur in the Páramo (Luteyn 1999), of which only 18 pass the high alpine-like criterion used here. Similarly, Hedberg (1961) states that 81% of Afroalpine species are endemic. Endemic in this context might easily be misinterpreted as endemic to the Afroalpine, however it instead means endemic to mountains of (eastern) Africa and while the Afroalpine does harbour a distinct set of species, many of these have a wider altitudinal range. The author would argue that, by distinguishing true alpine-like conditions from those at lower elevations and by identifying clades endemic to each, one can better understand the evolutionary history of true tropical alpine-like plants.

Despite the author’s use of the available phylogenetic data, the analysis presented here is still limited by uncertainty in discerning *in situ* alpine speciation and colonisation from lower elevations in the same area. The Andean dataset used for the estimation of species origins is particularly ridden with uncertainty as most published phylogenies of Andean Páramo plants are not well enough sampled or resolved to code species unambiguously. Additionally, the definition of Páramo by many authors is wide, often including all alpine-like or otherwise cool temperate areas without forest as low as 2800 m (Madriñán et al. 2013). It is possible that even more tropical alpine-like plants might have originated at lower elevations than inferred here (see also discussion in Hughes and Eastwood 2006; Sklenář et al. 2011; Madriñán et al. 2013). Many tropical alpine-like species and lineages in general have widespread geographical and altitudinal distributions in their respective areas and, for many of those, there is currently a lack of sufficiently sampled and/or resolved phylogenetic data necessary to code them accurately. Nevertheless, the overall patterns revealed in these analyses are clear and likely to be robust to the future addition of more fine-grained data for individual plant groups.

## Supplementary materials

Supplementary material 1 Detailed examples on how the coding was done Data type: methods This is an open access article distributed under the terms of the Creative Commons Attribution License (CC BY 4.0), which permits unrestricted use, distribution, and reproduction in any medium, provided the original author and source are credited.Author: Gehrke B

Supplementary material 2 List of genera investigated for the analysis including information on generic distribution, coding and references to the literature used Data type: species data This is an open access article distributed under the terms of the Creative Commons Attribution License (CC BY 4.0), which permits unrestricted use, distribution, and reproduction in any medium, provided the original author and source are credited.Author: Gehrke B

Supplementary material 3 Location of the tropical alpine-like climate regions in the Tropics Data type: occurence This is an open access article distributed under the terms of the Creative Commons Attribution License (CC BY 4.0), which permits unrestricted use, distribution, and reproduction in any medium, provided the original author and source are credited.Author: Gehrke B
