# Supplementary material for: Staying cool: preadaptation to temperate climates required for colonising tropical alpine-like environments
Source: PhytoKeys. 2018 Apr 17;(96):111–25. doi: 10.3897/phytokeys.96.13353 (PMC5915394; doi:10.3897/phytokeys.96.13353)
Supplement: Supplementary material 1 — Detailed examples on how the coding was done [file phytokeys-96-111-s003.pdf]

Supplement Figures 3-6: Location of the tropical alpine-like climate regions in the Tropics. Maps using a Mercator projection of the world with shaded relief and colored height based on Shuttle Radar Topography Mission (SRTM) data with 1 arc second resolution. Credit: NASA/JPL/NIMA downloaded from http://photojournal.jpl.nasa.gov/catalog/PIA03395.

In red regions were areas above 3800 m occur. Not drawn to scale due to the low extend of actual area above 3800m.

Supplement Figure 3: Tropical alpine-like areas in the Northern Andean Páramo

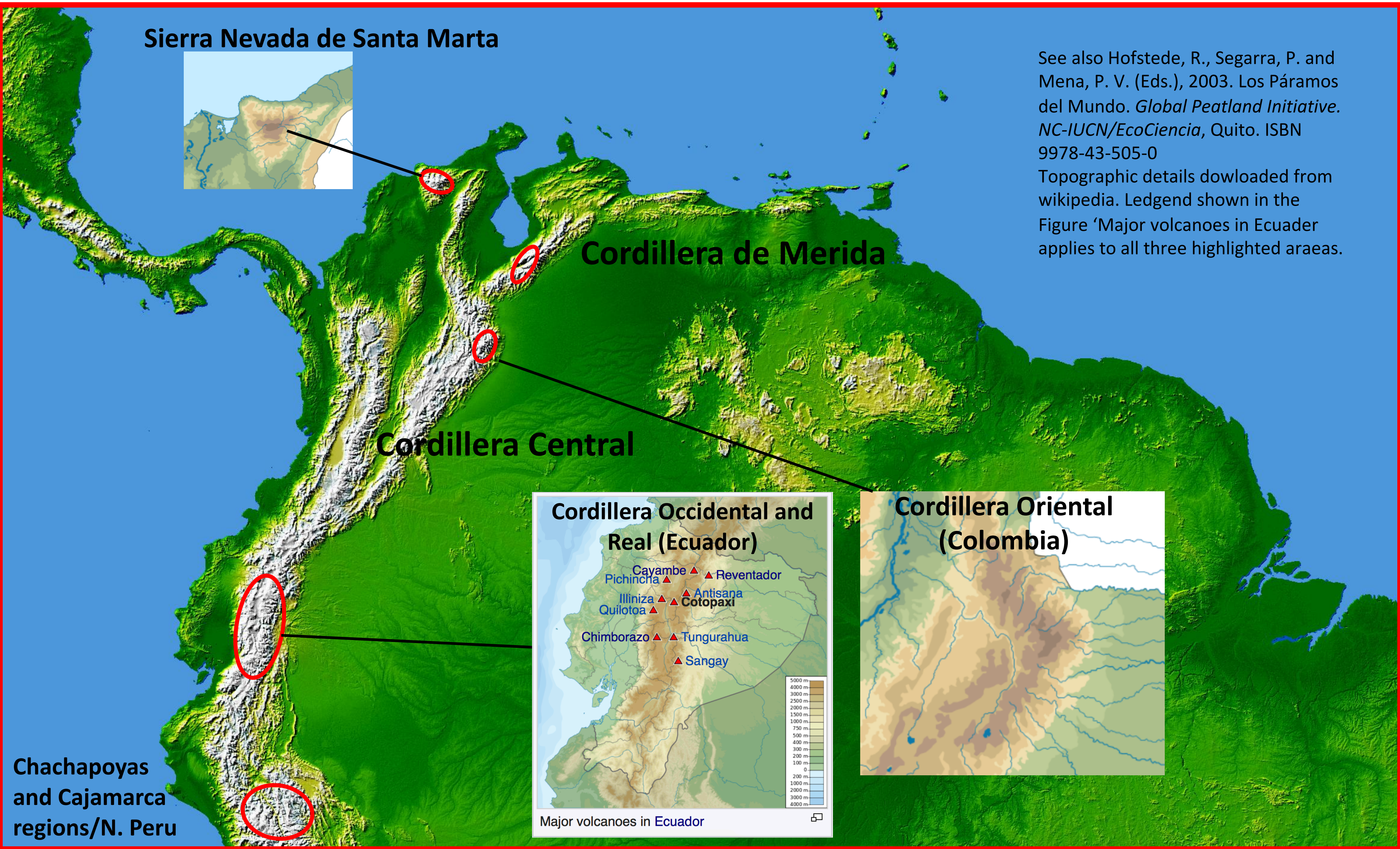

See also Hofstede, R., Segarra, P. and Mena, P. V. (Eds.), 2003. Los Páramos del Mundo. *Global Peatland Initiative. NC-IUCN/EcoCiencia*, Quito. ISBN 9978-43-505-0

Topographic details dowloaded from wikipedia. Ledgend shown in the Figure 'Major volcanoes in Ecuader applies to all three highlighted araeas.

Supplement Figure 4: Tropical alpine-like areas (Afroalpine) in Africa

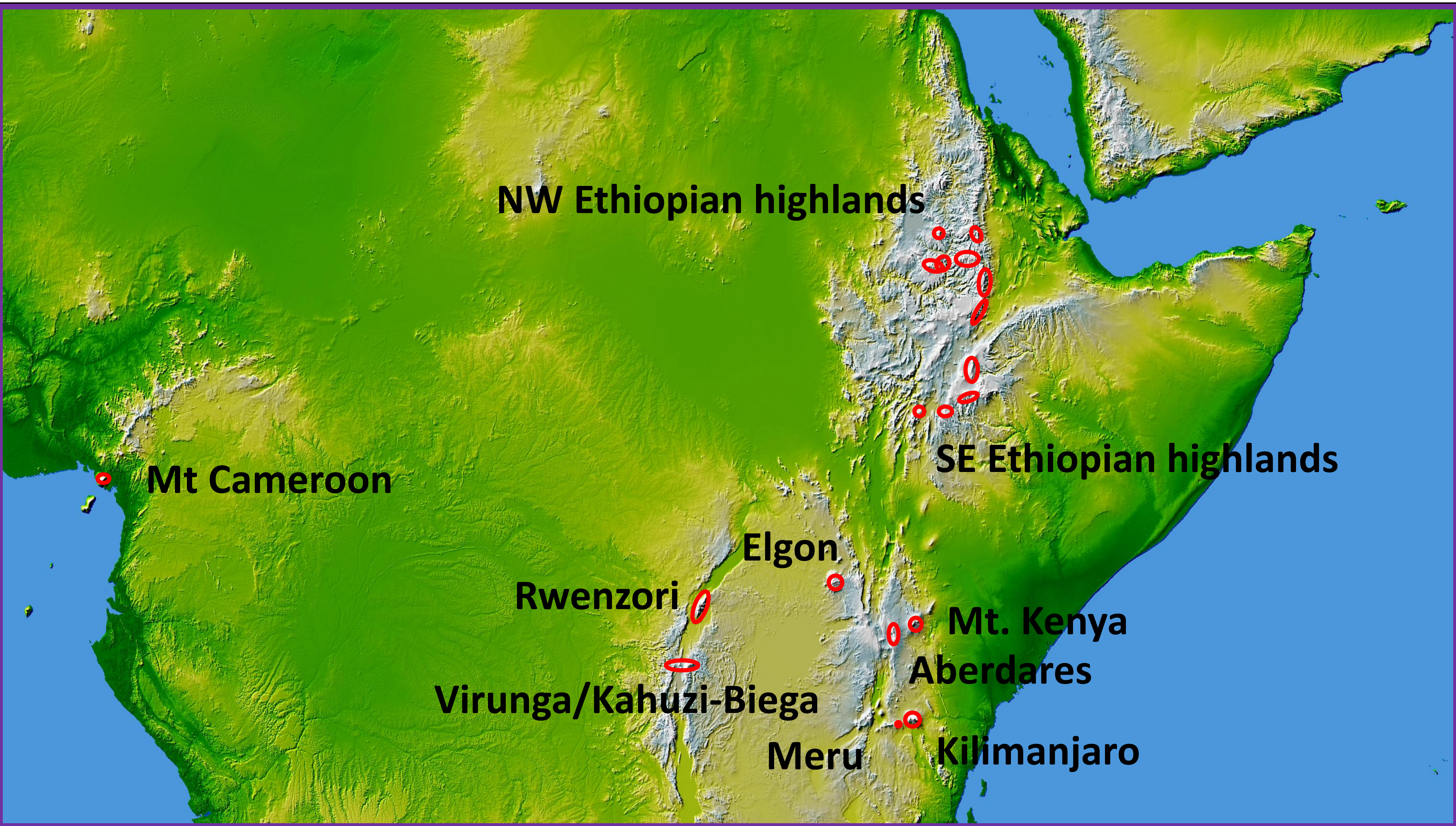

Supplement Figure 5: Tropical alpine-like regions in Asian New Guinea

about 45 peaks that rise above 3800 m exist on New Guinea

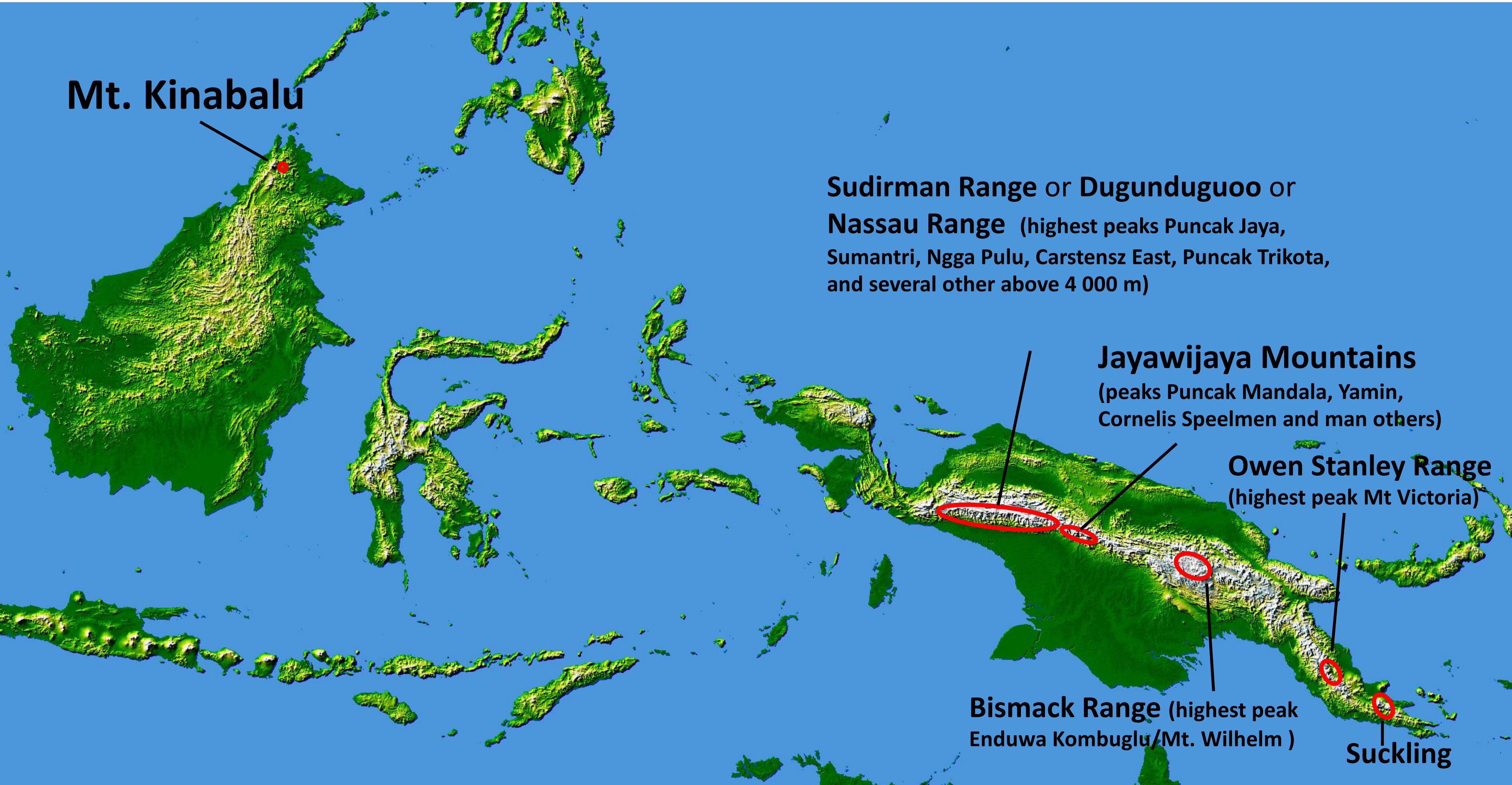

Supplement Figure 6: Tropical alpine-like part of the Hawaiian Islands

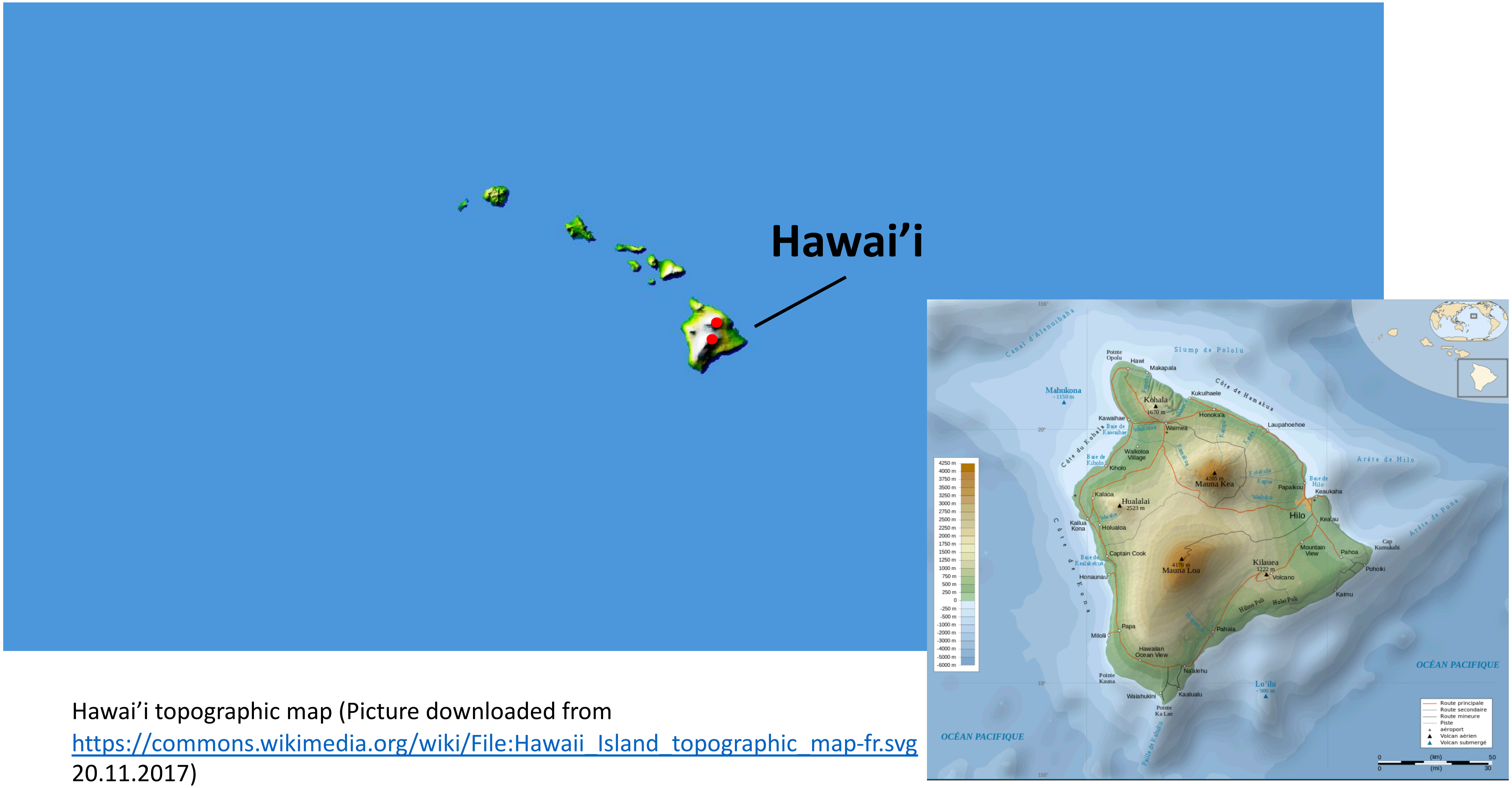

Hawai’i topographic map (Picture downloaded from https://commons.wikimedia.org/wiki/File:Hawaii_Island_topographic_map-fr.svg 20.11.2017)
